# Citation analysis of the most influential ependymoma research articles illustrates improved knowledge of the molecular biology of ependymoma

**DOI:** 10.1007/s10143-021-01579-1

**Published:** 2021-10-06

**Authors:** Nolan J. Brown, Bayard Wilson, Brian V. Lien, Alexander Himstead, Ali R. Tafreshi, Shane Shahrestani, Jack Birkenbeuel, Katelynn Tran, David Horton, Anushka Paladugu, Lydia R. Kirillova, Chen Yi Yang, Seth C. Ransom, Ronald Sahyouni, Isaac Yang

**Affiliations:** 1 Department of Neurological Surgery, University of California, Irvine, CA USA; 2 Department of Neurological Surgery, University of California, Los Angeles, CA USA; 3 Department of Neurological Surgery, Geisinger Health System, Danville, PA USA; 4 Medical Scientist Training Program, California Institute of Technology, Pasadena, CA USA; 5 Keck School of Medicine of USC, Los Angeles, CA USA; 6 College of Medicine, University of Arkansas for Medical Sciences, Little Rock, AR USA; 7 Department of Neurological Surgery, University of California, San Diego, La Jolla, CA USA; 8 Department of Neurosurgery, Los Angeles (UCLA), Los Angeles, CA USA; 9 Head and Neck Surgery, Los Angeles (UCLA), Los Angeles, CA USA; 10 Radiation Oncology, Los Angeles (UCLA), Los Angeles, CA USA; 11 Jonsson Comprehensive Cancer Center, Los Angeles (UCLA), Los Angeles, CA USA; 12 Los Angeles Biomedical Research Institute, Los Angeles (UCLA), Los Angeles, CA USA; 13 Harbor-UCLA Medical Center, Los Angeles (UCLA), Los Angeles, CA USA; 14 David Geffen School of Medicine, Los Angeles (UCLA), Los Angeles, CA USA

**Keywords:** Ependymoma, Ependymal tumors, Citation analysis, Bibliometric analysis

## Abstract

The history of academic research on ependymoma is expansive. This review summarizes its history with a bibliometric analysis of the 100 most cited articles on ependymoma. In March 2020, we queried the Web of Science database to identify the most cited articles on ependymoma using the terms “ependymoma” or “ependymal tumors,” yielding 3145 publications. Results were arranged by the number of times each article was cited in descending order. The top 100 articles spanned across nearly a century; the oldest article was published in 1924, while the most recent was in 2017. These articles were published in 35 unique journals, including a mix of basic science and clinical journals. The three institutions with the most papers in the top 100 were St. Jude Children’s Research Hospital (16%), the University of Texas MD Anderson Cancer Center (6%), and the German Cancer Research Center (5%). We analyzed the publications that may be considered the most influential in the understanding and treatment management of ependymoma. Studies focused on the molecular classification of ependymomas were well-represented among the most cited articles, reflecting the field’s current area of focus and its future directions. Additionally, this article also offers a reference for further studies in the ependymoma field.

## Introduction

Ependymomas are rare primary tumors of the central nervous system (CNS) that affect both children and adults [40, 42]. The 2016 World Health Organization (WHO) Classification of Tumors of the CNS categorizes them into four subtypes: subependymoma and myxopapillary ependymoma (grade I), ependymoma (grade II), ependymoma RELA (v-rel avian reticuloendotheliosis viral oncogene homolog A) fusion-positive (grade II or III), and anaplastic ependymoma (grade III) [12, 58]. Ependymomas are more commonly found in children and at this age more likely to be located intracranially and harbor more aggressive molecular variants, leading to worse overall survival (OS) when compared to adult variants [6, 14, 28, 30, 55].

Ependymomas have been extensively studied with respect to molecular subtyping, prognostication, and clinical outcomes [4, 8, 14, 30, 56, 58]. Therapy focuses on strategic surgical approaches to achieve gross total resection (GTR), and conformal radiation therapy (CRT) is the most common adjunctive treatment [6, 20, 21, 24, 38, 49]. Chemotherapy has been studied primarily in children under 3 years of age due to their susceptibility to radiotherapy-induced neurotoxicity. However, chemotherapy has failed to demonstrate improved outcomes compared to CRT [53]. At present, several clinical trials are underway scrutinizing promising neoadjuvant chemotherapeutic strategies [13, 32, 53]. Presently, effective treatment of ependymoma requires a multi-modal approach, involving an interdisciplinary team of neurosurgeons, neurologists, oncologists, radiologists, and primary care physicians, among others [14, 29]. Prognosis varies by type and location; pediatric ependymomas are more commonly intracranial with a 10-year estimated survival rate of 13–50%, while adult ependymomas have a predilection for the spine and have a 5-year survival rate of 67 to 85% and a 10-year survival rate of 72% [14, 30, 37]. In both populations, complete resection is the most consistent factor correlated with improved outcomes [6, 14, 37].

Given the diversity, volume, and interdisciplinary nature of ependymoma research, a bibliometric analysis focused on the history, recent developments, and trajectory of research can help frame our current understanding of the disease [3, 17]. The objective of this study is to analyze the most influential articles on ependymoma and identify the most relevant clinical problems in the field to guide further investigation. While bibliometric analyses have been published for neoplastic lesions of the brain [2, 3, 7, 17, 25] and for the spinal cord [1, 9, 10], no such investigation currently exists for ependymoma.

## Methods

On March 22, 2020, we performed a title-specific search of the Thomson Reuters Web of Science (WoS) database (Thomson Reuters, NY, USA) to identify the most cited articles on ependymoma. We used “ependymoma” or “ependymal tumors” as our query term for the years 1900 to 2020 selecting the “all databases” option. The results were arranged according to the number of times each article was cited in descending order. To avoid the subjective exclusion of studies from our analysis, all papers from our query were included if they were ranked 1 to 100 in terms of number of total citations. The following variables were extracted: rank of article by total citations, rank of article by average citations per year, first and last author, title of article, publication, year, total citations for each article, average citations per year for each article, article country of origin, and institution of the first author. In cases of co-first authorship, country and institution of the author listed first were used [3, 7, 17, 31]. The average citations per year for each article was calculated as previously described [10].

We categorized the articles as either clinical, basic science, or literature review. Articles were independently classified by LRK and DH and reexamined by BVL and AP. Any inconsistencies were resolved by discussion with the senior author (IY) after careful review of full-text articles. Studies that were primarily focused on basic tumor biology or molecular classification of ependymoma were classified as basic science (e.g., involving genome sequencing) as described previously [25]. Studies that were patient-focused and reported outcomes were classified as clinical, which included histopathological studies [25].

## Results

Our query yielded 3145 publications on ependymoma. The top 100 most cited articles were selected for review based on overall citation count and are shown in Tables 1 and 2, organized by total citations and average citations per year, respectively. These articles were published between 1924 and 2017. They have been cited a collective 11,640 times, averaging 116.4 citations per article (Table 1). The top 10 articles on the list were published between 1977 and 2015 and averaged 304 total citations (standard deviation [SD], 106.5; range, 206–551) (Table 1).

**Table 1:** Top 100 cited articles on ependymoma by citation number

Rank by total citations	Rank by average citations per year	Title	Authors (first/last)	Journal title	Publication year	Total citations	Average citations per year	Country	Type of study
1	3	Radial glia cells are candidate stem cells of ependymoma	Taylor MD, Gilbertson RJ	Cancer Cell	2005	551	34.44	USA	Basic science
2	8	Intramedullary ependymoma of the spinal cord	Mccormick PC, Stein BM	Journal of Neurosurgery	1990	453	14.61	USA	Clinical
3	1	Molecular classification of ependymal tumors across all CNS compartments, histopathological grades, and age groups	Pajtler KW, Pfister SM	Cancer Cell	2015	321	53.5	Germany	Basic science
4	33	Ependymoma: follow-up-study of 101 cases	Mork SJ, Loken AC	Cancer	1977	290	6.59	Norway	Clinical
5	2	C11orf95-RELA fusions drive oncogenic Nf-kappa B signalling in ependymoma	Parker M, Gilbertson RJ	Nature	2014	272	38.86	USA	Basic science
6	6	Conformal radiotherapy after surgery for paediatric ependymoma: a prospective study	Merchant TE, Sanford RA	Lancet Oncology	2009	258	21.5	USA	Clinical
7	4	Delineation of two clinically and molecularly distinct subgroups of posterior fossa ependymoma	Witt H, Pfister SM	Cancer Cell	2011	244	24.4	Germany	Basic science
8	38	Myxopapillary ependymoma: a clinicopathologic and immunocytochemical study of 77 cases	Sonneland PR, Onofrio BM	Cancer	1985	226	6.28	USA	Clinical
9	7	Cross-species genomics matches driver mutations and cell compartments to model ependymoma	Johnson RA, Gilbertson RJ	Nature	2010	219	19.91	USA	Basic science
10	25	Natural simian-virus-40 strains are present in human choroid-plexus and ependymoma tumors	Lednicky JA, Butel JS	Virology	1995	206	7.92	USA	Basic science
11	12	Preliminary results from a phase II trial of conformal radiation therapy and evaluation of radiation-related CNS effects for pediatric patients with localized ependymoma	Merchant TE, Sanford RA	Journal of Clinical Oncology	2004	201	11.82	USA	Clinical
12	40	The prognostic-significance of postoperative residual tumor in ependymoma	Healey EA, Tarbell NJ	Neurosurgery	1991	184	6.13	USA	Clinical
13	21	Postoperative chemotherapy without irradiation for ependymoma in children under 5 years of age: a multicenter trial of the French society of pediatric oncology	Grill J, Kalifa C	Journal of Clinical Oncology	2001	173	8.65	France	Clinical
14	14	Identification of tumor-specific molecular signatures in intracranial ependymoma and association with clinical characteristics	Modena P, Sozzi G	Journal of Clinical Oncology	2006	160	10.67	Italy	Basic science
15	15	Identification of gains on 1q and epidermal growth factor receptor overexpression as independent prognostic markers in intracranial ependymoma	Mendrzyk F, Lichter P	Clinical Cancer Research	2006	156	10.4	Germany	Basic science
16	29	Molecular genetic analysis of ependymal tumors: Nf2 mutations and chromosome 22q loss occur preferentially in intramedullary spinal ependymomas	Ebert C, Von Deimling A	American Journal of Pathology	1999	154	7	USA	Basic science
17	44	Treatment of intracranial ependymomas of children: review of a 15-year experience	Rousseau P, Rey A	International Journal of Radiation Oncology Biology Physics	1994	154	5.7	France	Clinical
18	34	Expression of vascular endothelial growth factor and its receptors in the anaplastic progression of astrocytoma, oligodendroglioma, and ependymoma	Chan AS, Chung LP	American Journal of Surgical Pathology	1998	151	6.57	Hong Kong	Basic science
19	63	Improved survival in cases of intracranial ependymoma after radiation-therapy: late report and recommendations	Salazar OM, Aygun C	Journal of Neurosurgery	1983	150	3.95	USA	Clinical
20	94	A metastasizing ependymoma of the cauda equina	Weiss, L	Cancer	1955	148	2.24	USA	Clinical
21	41	Analyses of prognostic factors in a retrospective review of 92 children with ependymoma: Italian pediatric neuro-oncology group	Perilongo G, Madon E	Medical and Pediatric Oncology	1997	144	6	Italy	Clinical
22	75	Symptomatic subependymoma: report of 21 cases with review of literature	Scheithauer BW	Journal of Neurosurgery	1978	141	3.28	USA	Clinical
23	16	Primary postoperative chemotherapy without radiotherapy for intracranial ependymoma in children: the UKCCSG/SIOP prospective study	Grundy RG, Machin D	Lancet Oncology	2007	139	9.93	UK	Clinical
24	74	Differential-diagnosis of chordoma, chondroid, and ependymal tumors as aided by anti-intermediate filament antibodies	Miettinen M, Virtanen I	American Journal of Pathology	1983	129	3.39	Finland	Clinical
25	57	Intracranial ependymoma: long-term results of a policy of surgery and radiotherapy	Vanuytsel LJ, Brada M	International Journal of Radiation Oncology Biology Physics	1992	128	4.41	UK	Clinical
26	32	Spinal cord ependymoma: radical surgical resection and outcome	Hanbali F, Gokaslan ZL	Neurosurgery	2002	127	6.68	USA	Clinical
27	17	Proton radiotherapy for childhood ependymoma: initial clinical outcomes and dose comparisons	Macdonald SM, Yock T	International Journal of Radiation Oncology Biology Physics	2008	126	9.69	USA	Clinical
28	47	Ependymoma: results, prognostic factors and treatment recommendations	Mclaughlin MP, Million RR	International Journal of Radiation Oncology Biology Physics	1998	126	5.48	USA	Clinical
29	69	Postoperative radiotherapy of intracranial ependymoma in pediatric and adult patients	Shaw EG, Earle JD	International Journal of Radiation Oncology Biology Physics	1987	126	3.71	USA	Clinical
30	11	Histopathological grading of pediatric ependymoma: reproducibility and clinical relevance in European trial cohorts	Ellison DW, Grundy RG	Journal of Negative Results in Biomedicine	2011	123	12.3	USA	Clinical
31	35	Erbb receptor signaling promotes ependymoma cell proliferation and represents a potential novel therapeutic target for this disease	Gilbertson RJ Ellison DW	Clinical Cancer Research	2002	123	6.47	USA	Basic science
32	26	Monomorphous angiocentric glioma: a distinctive epileptogenic neoplasm with features of infiltrating astrocytoma and ependymoma	Wang M, Burger PC	Journal of Neuropathology and Experimental Neurology	2005	121	7.56	USA	Clinical
33	46	A multi-institutional retrospective study of intracranial ependymoma in children: identification of risk factors	Horn B, Russo C	Journal of Pediatric Hematology Oncology	1999	121	5.5	USA	Clinical
34	61	Identification of a germ-line mutation in the p53 gene in a patient with an intracranial ependymoma	Metzger AK, Cogen PH	Proceedings of The National Academy of Sciences of the United States of America	1991	120	4	USA	Clinical
35	13	Molecular staging of intracranial ependymoma in children and adults	Korshunov A, Pfister SM	Journal of Clinical Oncology	2010	119	10.82	Germany	Basic science
36	45	Combined postoperative irradiation and chemotherapy for anaplastic ependymomas in childhood: results of the German prospective trials hit 88/89 and hit 91	Timmermann B Bamberg M	International Journal of Radiation Oncology Biology Physics	2000	118	5.62	Germany	Clinical
37	65	Histologic prognostic factors in ependymoma	Schiffer D, Tribolo A	Childs Nervous System	1991	116	3.87	Italy	Clinical
38	19	Incidence patterns for ependymoma: a surveillance, epidemiology, and end results study clinical article	Mcguire CS, Fisher PG	Journal of Neurosurgery	2009	112	9.33	USA	Clinical
39	59	Adjuvant chemotherapy of childhood posterior fossa ependymoma: cranio-spinal irradiation with or without adjuvant CCNU, vincristine, and prednisone: a children’s cancer group study	Evans AE, Finlay JL	Medical and Pediatric Oncology	1996	106	4.24	USA	Clinical
40	73	Postoperative radiotherapy in the management of spinal cord ependymoma	Whitaker SJ, Brada M	Journal of Neurosurgery	1991	105	3.5	UK	Clinical
41	82	Subcutaneous sacrococcygeal myxopapillary ependymoma: a clinicopathologic study of 32 cases	Helwig EB, Stern JB	American Journal of Clinical Pathology	1984	102	2.76	USA	Clinical
42	22	Pediatric ependymoma: biological perspectives	Kilday JP, Grundy R	Molecular Cancer Research	2009	101	8.42	UK	Review
43	93	Extra-spinal ependymomas: report of 3 cases	Morantz RA, Masterson BJ	Journal of Neurosurgery	1979	99	2.36	USA	Clinical
44	51	Chromosomal abnormalities subdivide ependymal tumors into clinically relevant groups	Hirose Y, Feuerstein BG	American Journal of Pathology	2001	98	4.9	USA	Basic science
45	60	Adjuvant chemotherapy for the treatment of intracranial ependymoma of childhood	Needle MN, Phillips PC	Cancer	1997	97	4.04	USA	Clinical
46	27	A retrospective study of surgery and reirradiation for recurrent ependymoma	Merchant TE, Sanford RA	International Journal of Radiation Oncology Biology Physics	2008	96	7.38	USA	Clinical
47	96	Secretory ependymoma of filum terminale	Miller CA Torack RA	Acta Neuropathologica	1970	95	1.86	USA	Clinical
48	95	Delayed distant metastasis from a subcutaneous sacrococcygeal ependymoma: case report, with tissue-culture, ultrastructural observations and review of literature	Wolff M, Duby MM	Cancer	1972	93	1.9	USA	Review
49	36	Ependymoma	Reni M, Vecht C	Critical Reviews in Oncology Hematology	2007	90	6.43	Italy	Review
50	5	The current consensus on the clinical management of intracranial ependymoma and its distinct molecular variants	Pajtler KW, Taylor MD	Acta Neuropathologica	2017	89	22.25	Germany	Review
51	97	Is subependymoma (subependymal glomerate astrocytoma) an astrocytoma or ependymoma: comparative ultrastructural and tissue-culture study	Fu YS, Young HF	Cancer	1974	86	1.83	USA	Clinical
52	71	Anaplastic ependymoma: treatment of pediatric patients with or without craniospinal radiation therapy	Merchant TE, Leibel SA	Journal of Neurosurgery	1997	85	3.54	USA	Clinical
53	87	Ependymal and choroid-plexus tumors: cytokeratin and GFAP expression	Mannoji H, Becker LE	Cancer	1988	84	2.55	Canada	Clinical
54	37	Predicting change in academic abilities after conformal radiation therapy for localized ependymoma	Conklin HM, Merchant TE	Journal of Clinical Oncology	2008	83	6.38	USA	Clinical
55	31	Both location and age predict survival in ependymoma: a seer study	Mcguire CS, Fisher PG	Pediatric Blood & Cancer	2009	81	6.75	USA	Clinical
56	72	Treatment of intracranial ependymoma by surgery alone	Hukin J, Allen J	Pediatric Neurosurgery	1998	81	3.52	USA	Clinical
57	24	Identification of microRNAs as potential prognostic markers in ependymoma	Costa FF Soares MB	Plos One	2011	80	8	USA	Basic science
58	39	Biological background of pediatric medulloblastoma and ependymoma: a review from a translational research perspective	De Bont JM, Pieters R	Neuro-Oncology	2008	80	6.15	Netherlands	Review
59	48	Spinal myxopapillary ependymoma outcomes in patients treated with surgery and radiotherapy at MD Anderson Cancer Center	Akyurek S, Woo SY	Journal of Neuro-Oncology	2006	80	5.33	USA	Clinical
60	84	Ependymoma: internal correlations among pathological signs: the anaplastic variant	Schiffer D, Vigliani MC	Neurosurgery	1991	80	2.67	Italy	Clinical
61	99	Ependymoma of the brain: pathologic aspects	Svien HJ, Craig WM	Neurology	1953	80	1.18	USA	Review
62	85	The role of prophylactic spinal irradiation in localized intracranial ependymoma	Vanuytsel L, Brada M	International Journal of Radiation Oncology Biology Physics	1991	79	2.63	UK	Clinical
63	20	A prognostic gene expression signature in infratentorial ependymoma	Wani K, Aldape K	Acta Neuropathologica	2012	78	8.67	USA	Basic science
64	42	Multifactorial analysis of predictors of outcome in pediatric intracranial ependymoma	Ridley L, Grundy RG	Neuro-Oncology	2008	78	6	UK	Clinical
65	52	Radiation dosimetry predicts IQ after conformal radiation therapy in pediatric patients with localized ependymoma	Merchant TE, Mulhern RK	International Journal of Radiation Oncology Biology Physics	2005	78	4.88	USA	Clinical
66	53	Ependymoma: new therapeutic approaches including radiation and chemotherapy	Merchant TE, Fouladi M	Journal of Neuro-Oncology	2005	78	4.88	USA	Clinical
67	81	Intracranial ependymoma long-term outcome, patterns of failure	Kovalic JJ, Roth KA	Journal of Neuro-Oncology	1993	78	2.79	USA	Clinical
68	88	Intracranial ependymoma and subependymoma: MR manifestations	Spoto GP, Solomon M	American Journal of Neuroradiology	1990	78	2.52	USA	Clinical
69	18	Proton radiotherapy for pediatric central nervous system ependymoma: clinical outcomes for 70 patients	Macdonald SM, Yock TI	Neuro-Oncology	2013	76	9.5	USA	Clinical
70	50	Human telomere reverse transcriptase expression predicts progression and survival in pediatric intracranial ependymoma	Tabori U, Hawkins C	Journal of Clinical Oncology	2006	76	5.07	Canada	Clinical
71	100	A study of tumors arising from ependymal cells	Bailey P	Archives of Neurology And Psychiatry	1924	75	0.77	USA	Clinical
72	89	Intracranial ependymoma in children: analysis of prognostic factors	Chiu JK, Shallenberger R	Journal of Neuro-Oncology	1992	73	2.52	USA	Clinical
73	66	Ependymoma in childhood: prognostic factors, extent of surgery, and adjuvant therapy	van Veelen-Vincent, ML, Renier D	Journal of Neurosurgery	2002	72	3.79	Netherlands	Clinical
74	28	An integrated in vitro and in vivo high-throughput screen identifies treatment leads for ependymoma	Atkinson JM, Gilbertson RJ	Cancer Cell	2011	71	7.1	USA	Basic science
75	67	Influence of tumor grade on time to progression after irradiation for localized ependymoma in children	Merchant TE, Kun LE	International Journal of Radiation Oncology Biology Physics	2002	71	3.74	USA	Clinical
76	68	Preliminary results from a phase II trial of conformal radiation therapy for pediatric patients with localized low-grade astrocytoma and ependymoma	Merchant TE, Kun LE	International Journal of Radiation Oncology Biology Physics	2002	71	3.74	USA	Clinical
77	58	The high incidence of tumor dissemination in myxopapillary ependymoma in pediatric patients: report of five cases and review of the literature	Fassett DR, Kestle JRW	Journal of Neurosurgery	2005	70	4.38	USA	Clinical
78	49	Differential expression and prognostic significance of sox genes in pediatric medulloblastoma and ependymoma identified by microarray analysis	De Bont JM, Pieters R	Neuro-Oncology	2008	69	5.31	Netherlands	Basic science
79	62	A multicenter study of the prognosis and treatment of adult brain ependymal tumors	Reni M, Villa E	Cancer	2004	68	4	Italy	Clinical
80	54	Ependymoma gene expression profiles associated with histological subtype, proliferation, and patient survival	Lukashova-Von Zangen I, Roggendorf W	Acta Neuropathologica	2007	66	4.71	Germany	Basic science
81	64	Ki-67 immunolabeling index is an accurate predictor of outcome in patients with intracranial ependymoma	Wolfsberger S, Hainfellner J	American Journal of Surgical Pathology	2004	66	3.88	Austria	Clinical
82	9	Clinical evidence of variable proton biological effectiveness in pediatric patients treated for ependymoma	Peeler CR, Grosshans DR	Radiotherapy and Oncology	2016	65	13	USA	Clinical
83	10	Therapeutic impact of cytoreductive surgery and irradiation of posterior fossa ependymoma in the molecular era: a retrospective multicohort analysis	Ramaswamy V, Taylor MD	Journal of Clinical Oncology	2016	65	13	Canada	Clinical
84	43	Primary postoperative chemotherapy without radiotherapy for treatment of brain tumours other than ependymoma in children under 3 years: results of the first UKCCSG/SIOP CNS 9204 trial	Grundy RG, Machin D	European Journal of Cancer	2010	65	5.91	UK	Clinical
85	78	Chromosome arm 6q loss is the most common recurrent autosomal alteration detected in primary pediatric ependymoma	Reardon DA, Look AT	Genes Chromosomes & Cancer	1999	65	2.95	USA	Basic science
86	90	MR characteristics of histopathologic subtypes of spinal ependymoma	Kahan H, Bruce JH	American Journal of Neuroradiology	1996	63	2.52	USA	Clinical
87	98	Melanin as a component of cerebral gliomas: melanotic cerebral ependymoma	Mccloskey JJ, Blacker HM	Cancer	1976	63	1.4	USA	Clinical
88	30	Survival benefit for pediatric patients with recurrent ependymoma treated with reirradiation	Bouffet E, Tabori U	International Journal of Radiation Oncology Biology Physics	2012	62	6.89	Canada	Clinical
89	56	Outcome for young children newly diagnosed with ependymoma, treated with intensive induction chemotherapy followed by myeloablative chemotherapy and autologous stem cell rescue	Zacharoulis S, Finlay J	Pediatric Blood & Cancer	2007	62	4.43	USA	Clinical
90	70	Hyperfractionated radiotherapy and chemotherapy for childhood ependymoma: final results of the first prospective AIEOP (Associazione Italiana di Ematologia-Oncologia Pediatrica) study	Massimino M, Madon E	International Journal of Radiation Oncology Biology Physics	2004	62	3.65	Italy	Clinical
91	76	Postoperative radiotherapy for intracranial ependymoma: analysis of prognostic factors and patterns of failure	Oya N, Hiraoka M	Journal of Neuro-Oncology	2002	62	3.26	Japan	Clinical
92	92	A high-dose busulfan-thiotepa combination followed by autologous bone marrow transplantation in childhood recurrent ependymoma: a phase-II study	Grill J, Hartmann O	Pediatric Neurosurgery	1996	61	2.44	France	Clinical
93	83	Clinicopathologic study of 61 patients with ependymoma including MIB-1 immunohistochemistry	Prayson RA	Annals of Diagnostic Pathology	1999	60	2.73	USA	Clinical
94	86	Survival following intensive chemotherapy with bone marrow reconstitution for children with recurrent intracranial ependymoma: a report of the children's cancer group	Mason WP, Finlay JL	Journal of Neuro-Oncology	1998	60	2.61	USA	Clinical
95	55	Central nervous system tumors with ependymal features: a broadened spectrum of primarily ependymal differentiation?	Lehman NL	Journal of Neuropathology And Experimental Neurology	2008	59	4.54	USA	Review
96	77	Astroblastoma: radiologic-pathologic correlation and distinction from ependymoma	Port JD, Pomper MG	American Journal of Neuroradiology	2002	59	3.11	USA	Clinical
97	79	Stereotactic radiosurgery for recurrent ependymoma	Stafford SL, Schomberg PJ	Cancer	2000	59	2.81	USA	Clinical
98	80	Pediatric low-grade and ependymal spinal cord tumors	Merchant TE, Kun LE	Pediatric Neurosurgery	2000	59	2.81	USA	Clinical
99	91	Tanycytic ependymoma	Langford LA, Barre GM	Ultrastructural Pathology	1997	59	2.46	USA	Clinical
100	23	Clinical, radiological, histological and molecular characteristics of paediatric epithelioid glioblastoma	Broniscer A, Ellison DW	Neuropathology and Applied Neurobiology	2014	58	8.29	USA	Clinical

**Table 2:** Top 100 cited articles on ependymoma by average citations per year

Rank by total citations	Rank by average citations per year	Title	Authors (first and last)	Journal title	Publication year	Total citations	Average citations per year	Country	Type of study
3	1	Molecular classification of ependymal tumors across all CNS compartments, histopathological grades, and age groups	Pajtler KW, Pfister SM	Cancer Cell	2015	321	53.5	Germany	Basic science
5	2	C11orf95-RELA fusions drive oncogenic Nf-kappa B signalling in ependymoma	Parker M, Gilbertson RJ	Nature	2014	272	38.86	USA	Basic science
1	3	Radial glia cells are candidate stem cells of ependymoma	Taylor MD, Gilbertson RJ	Cancer Cell	2005	551	34.44	USA	Basic science
7	4	Delineation of two clinically and molecularly distinct subgroups of posterior fossa ependymoma	Witt H, Pfister SM	Cancer Cell	2011	244	24.4	Germany	Basic science
50	5	The current consensus on the clinical management of intracranial ependymoma and its distinct molecular variants	Pajtler KW, Taylor MD	Acta Neuropathologica	2017	89	22.25	Germany	Review
6	6	Conformal radiotherapy after surgery for paediatric ependymoma: a prospective study	Merchant TE, Sanford RA	Lancet Oncology	2009	258	21.5	USA	Clinical
9	7	Cross-species genomics matches driver mutations and cell compartments to model ependymoma	Johnson RA, Gilbertson RJ	Nature	2010	219	19.91	USA	Basic science
2	8	Intramedullary ependymoma of the spinal cord	Mccormick PC, Stein BM	Journal of Neurosurgery	1990	453	14.61	USA	Clinical
82	9	Clinical evidence of variable proton biological effectiveness in pediatric patients treated for ependymoma	Peeler CR, Grosshans DR	Radiotherapy and Oncology	2016	65	13	USA	Clinical
83	10	Therapeutic impact of cytoreductive surgery and irradiation of posterior fossa ependymoma in the molecular era: a retrospective multicohort analysis	Ramaswamy V, Taylor MD	Journal of Clinical Oncology	2016	65	13	Canada	Clinical
30	11	Histopathological grading of pediatric ependymoma: reproducibility and clinical relevance in European trial cohorts	Ellison DW, Grundy RG	Journal of Negative Results in Biomedicine	2011	123	12.3	USA	Clinical
11	12	Preliminary results from a phase II trial of conformal radiation therapy and evaluation of radiation-related CNS effects for pediatric patients with localized ependymoma	Merchant TE, Sanford RA	Journal of Clinical Oncology	2004	201	11.82	USA	Clinical
35	13	Molecular staging of intracranial ependymoma in children and adults	Korshunov A, Pfister SM	Journal of Clinical Oncology	2010	119	10.82	Germany	Basic science
14	14	Identification of tumor-specific molecular signatures in intracranial ependymoma and association with clinical characteristics	Modena P, Sozzi G	Journal of Clinical Oncology	2006	160	10.67	Italy	Basic science
15	15	Identification of gains on 1q and epidermal growth factor receptor overexpression as independent prognostic markers in intracranial ependymoma	Mendrzyk F, Lichter P	Clinical Cancer Research	2006	156	10.4	Germany	Basic science
23	16	Primary postoperative chemotherapy without radiotherapy for intracranial ependymoma in children: the UKCCSG/SIOP prospective study	Grundy RG, Machin D	Lancet Oncology	2007	139	9.93	UK	Clinical
27	17	Proton radiotherapy for childhood ependymoma: initial clinical outcomes and dose comparisons	Macdonald SM, Yock T	International Journal of Radiation Oncology Biology Physics	2008	126	9.69	USA	Clinical
69	18	Proton radiotherapy for pediatric central nervous system ependymoma: clinical outcomes for 70 patients	Macdonald SM, Yock TI	Neuro-Oncology	2013	76	9.5	USA	Clinical
38	19	Incidence patterns for ependymoma: a surveillance, epidemiology, and end results study clinical article	Mcguire CS, Fisher PG	Journal of Neurosurgery	2009	112	9.33	USA	Clinical
63	20	A prognostic gene expression signature in infratentorial ependymoma	Wani K, Aldape K	Acta Neuropathologica	2012	78	8.67	USA	Basic science
13	21	Postoperative chemotherapy without irradiation for ependymoma in children under 5 years of age: a multicenter trial of the French society of pediatric oncology	Grill J, Kalifa C	Journal of Clinical Oncology	2001	173	8.65	France	Clinical
42	22	Pediatric ependymoma: biological perspectives	Kilday JP, Grundy R	Molecular Cancer Research	2009	101	8.42	UK	Review
100	23	Clinical, radiological, histological and molecular characteristics of paediatric epithelioid glioblastoma	Broniscer A, Ellison DW	Neuropathology and Applied Neurobiology	2014	58	8.29	USA	Clinical
57	24	Identification of microRNAs as potential prognostic markers in ependymoma	Costa FF Soares MB	Plos One	2011	80	8	USA	Basic science
10	25	Natural simian-virus-40 strains are present in human choroid-plexus and ependymoma tumors	Lednicky JA, Butel JS	Virology	1995	206	7.92	USA	Basic science
32	26	Monomorphous angiocentric glioma: a distinctive epileptogenic neoplasm with features of infiltrating astrocytoma and ependymoma	Wang M, Burger PC	Journal of Neuropathology and Experimental Neurology	2005	121	7.56	USA	Clinical
46	27	A retrospective study of surgery and reirradiation for recurrent ependymoma	Merchant TE, Sanford RA	International Journal of Radiation Oncology Biology Physics	2008	96	7.38	USA	Clinical
74	28	An integrated in vitro and in vivo high-throughput screen identifies treatment leads for ependymoma	Atkinson JM, Gilbertson RJ	Cancer Cell	2011	71	7.1	USA	Basic science
16	29	Molecular genetic analysis of ependymal tumors: Nf2 mutations and chromosome 22q loss occur preferentially in intramedullary spinal ependymomas	Ebert C, Von Deimling A	American Journal of Pathology	1999	154	7	USA	Basic science
88	30	Survival benefit for pediatric patients with recurrent ependymoma treated with reirradiation	Bouffet E, Tabori U	International Journal of Radiation Oncology Biology Physics	2012	62	6.89	Canada	Clinical
55	31	Both location and age predict survival in ependymoma: a seer study	Mcguire CS, Fisher PG	Pediatric Blood & Cancer	2009	81	6.75	USA	Clinical
26	32	Spinal cord ependymoma: radical surgical resection and outcome	Hanbali F, Gokaslan ZL	Neurosurgery	2002	127	6.68	USA	Clinical
4	33	Ependymoma: follow-up-study of 101 cases	Mork SJ, Loken AC	Cancer	1977	290	6.59	Norway	Clinical
18	34	Expression of vascular endothelial growth factor and its receptors in the anaplastic progression of astrocytoma, oligodendroglioma, and ependymoma	Chan AS, Chung LP	American Journal of Surgical Pathology	1998	151	6.57	Hong Kong	Basic science
31	35	Erbb receptor signaling promotes ependymoma cell proliferation and represents a potential novel therapeutic target for this disease	Gilbertson RJ Ellison DW	Clinical Cancer Research	2002	123	6.47	USA	Basic science
49	36	Ependymoma	Reni M, Vecht C	Critical Reviews in Oncology Hematology	2007	90	6.43	Italy	Review
54	37	Predicting change in academic abilities after conformal radiation therapy for localized ependymoma	Conklin HM, Merchant TE	Journal of Clinical Oncology	2008	83	6.38	USA	Clinical
8	38	Myxopapillary ependymoma: a clinicopathologic and immunocytochemical study of 77 cases	Sonneland PR, Onofrio BM	Cancer	1985	226	6.28	USA	Clinical
58	39	Biological background of pediatric medulloblastoma and ependymoma: a review from a translational research perspective	De Bont JM, Pieters R	Neuro-Oncology	2008	80	6.15	Netherlands	Review
12	40	The prognostic-significance of postoperative residual tumor in ependymoma	Healey EA, Tarbell NJ	Neurosurgery	1991	184	6.13	USA	Clinical
21	41	Analyses of prognostic factors in a retrospective review of 92 children with ependymoma: Italian Pediatric Neuro-oncology Group	Perilongo G, Madon E	Medical and Pediatric Oncology	1997	144	6	Italy	Clinical
64	42	Multifactorial analysis of predictors of outcome in pediatric intracranial ependymoma	Ridley L, Grundy RG	Neuro-Oncology	2008	78	6	UK	Clinical
84	43	Primary postoperative chemotherapy without radiotherapy for treatment of brain tumours other than ependymoma in children under 3 years: results of the first UKCCSG/SIOP CNS 9204 trial	Grundy RG, Machin D	European Journal of Cancer	2010	65	5.91	UK	Clinical
17	44	Treatment of intracranial ependymomas of children: review of a 15-year experience	Rousseau P, Rey A	International Journal of Radiation Oncology Biology Physics	1994	154	5.7	France	Clinical
36	45	Combined postoperative irradiation and chemotherapy for anaplastic ependymomas in childhood: results of the German prospective trials hit 88/89 and hit 91	Timmermann B Bamberg M	International Journal of Radiation Oncology Biology Physics	2000	118	5.62	Germany	Clinical
33	46	A multi-institutional retrospective study of intracranial ependymoma in children: identification of risk factors	Horn B, Russo C	Journal of Pediatric Hematology Oncology	1999	121	5.5	USA	Clinical
28	47	Ependymoma: results, prognostic factors and treatment recommendations	Mclaughlin MP, Million RR	International Journal of Radiation Oncology Biology Physics	1998	126	5.48	USA	Clinical
59	48	Spinal myxopapillary ependymoma outcomes in patients treated with surgery and radiotherapy at MD Anderson Cancer Center	Akyurek S, Woo SY	Journal of Neuro-Oncology	2006	80	5.33	USA	Clinical
78	49	Differential expression and prognostic significance of sox genes in pediatric medulloblastoma and ependymoma identified by microarray analysis	De Bont JM, Pieters R	Neuro-Oncology	2008	69	5.31	Netherlands	Basic science
70	50	Human telomere reverse transcriptase expression predicts progression and survival in pediatric intracranial ependymoma	Tabori U, Hawkins C	Journal of Clinical Oncology	2006	76	5.07	Canada	Clinical
44	51	Chromosomal abnormalities subdivide ependymal tumors into clinically relevant groups	Hirose Y, Feuerstein BG	American Journal of Pathology	2001	98	4.9	USA	Basic science
65	52	Radiation dosimetry predicts iq after conformal radiation therapy in pediatric patients with localized ependymoma	Merchant TE, Mulhern RK	International Journal of Radiation Oncology Biology Physics	2005	78	4.88	USA	Clinical
66	53	Ependymoma: new therapeutic approaches including radiation and chemotherapy	Merchant TE, Fouladi M	Journal of Neuro-Oncology	2005	78	4.88	USA	Clinical
80	54	Ependymoma gene expression profiles associated with histological subtype, proliferation, and patient survival	Lukashova-Von Zangen I, Roggendorf W	Acta Neuropathologica	2007	66	4.71	Germany	Basic science
95	55	Central nervous system tumors with ependymal features: a broadened spectrum of primarily ependymal differentiation?	Lehman NL	Journal of Neuropathology And Experimental Neurology	2008	59	4.54	USA	Review
89	56	Outcome for young children newly diagnosed with ependymoma, treated with intensive induction chemotherapy followed by myeloablative chemotherapy and autologous stem cell rescue	Zacharoulis S, Finlay J	Pediatric Blood & Cancer	2007	62	4.43	USA	Clinical
25	57	Intracranial ependymoma: long-term results of a policy of surgery and radiotherapy	Vanuytsel LJ, Brada M	International Journal of Radiation Oncology Biology Physics	1992	128	4.41	UK	Clinical
77	58	The high incidence of tumor dissemination in myxopapillary ependymoma in pediatric patients: report of five cases and review of the literature	Fassett DR, Kestle JRW	Journal of Neurosurgery	2005	70	4.38	USA	Clinical
39	59	Adjuvant chemotherapy of childhood posterior fossa ependymoma: cranio-spinal irradiation with or without adjuvant CCNU, vincristine, and prednisone: a children’s cancer group study	Evans AE, Finlay JL	Medical and Pediatric Oncology	1996	106	4.24	USA	Clinical
45	60	Adjuvant chemotherapy for the treatment of intracranial ependymoma of childhood	Needle MN, Phillips PC	Cancer	1997	97	4.04	USA	Clinical
34	61	Identification of a germ-line mutation in the p53 gene in a patient with an intracranial ependymoma	Metzger AK, Cogen PH	Proceedings of The National Academy of Sciences of the United States of America	1991	120	4	USA	Clinical
79	62	A multicenter study of the prognosis and treatment of adult brain ependymal tumors	Reni M, Villa E	Cancer	2004	68	4	Italy	Clinical
19	63	Improved survival in cases of intracranial ependymoma after radiation-therapy: late report and recommendations	Salazar OM, Aygun C	Journal of Neurosurgery	1983	150	3.95	USA	Clinical
81	64	Ki-67 immunolabeling index is an accurate predictor of outcome in patients with intracranial ependymoma	Wolfsberger S, Hainfellner J	American Journal of Surgical Pathology	2004	66	3.88	Austria	Clinical
37	65	Histologic prognostic factors in ependymoma	Schiffer D, Tribolo A	Childs Nervous System	1991	116	3.87	Italy	Clinical
73	66	Ependymoma in childhood: prognostic factors, extent of surgery, and adjuvant therapy	van Veelen-Vincent, ML, Renier D	Journal of Neurosurgery	2002	72	3.79	Netherlands	Clinical
75	67	Influence of tumor grade on time to progression after irradiation for localized ependymoma in children	Merchant TE, Kun LE	International Journal of Radiation Oncology Biology Physics	2002	71	3.74	USA	Clinical
76	68	Preliminary results from a phase II trial of conformal radiation therapy for pediatric patients with localized low-grade astrocytoma and ependymoma	Merchant TE, Kun LE	International Journal of Radiation Oncology Biology Physics	2002	71	3.74	USA	Clinical
29	69	Postoperative radiotherapy of intracranial ependymoma in pediatric and adult patients	Shaw EG, Earle JD	International Journal of Radiation Oncology Biology Physics	1987	126	3.71	USA	Clinical
90	70	Hyperfractionated radiotherapy and chemotherapy for childhood ependymoma: final results of the first prospective AIEOP (Associazione Italiana di Ematologia-Oncologia Pediatrica) study	Massimino M, Madon E	International Journal of Radiation Oncology Biology Physics	2004	62	3.65	Italy	Clinical
52	71	Anaplastic ependymoma: treatment of pediatric patients with or without craniospinal radiation therapy	Merchant TE, Leibel SA	Journal of Neurosurgery	1997	85	3.54	USA	Clinical
56	72	Treatment of intracranial ependymoma by surgery alone	Hukin J, Allen J	Pediatric Neurosurgery	1998	81	3.52	USA	Clinical
40	73	Postoperative radiotherapy in the management of spinal-cord ependymoma	Whitaker SJ, Brada M	Journal of Neurosurgery	1991	105	3.5	UK	Clinical
24	74	Differential-diagnosis of chordoma, chondroid, and ependymal tumors as aided by anti-intermediate filament antibodies	Miettinen M, Virtanen I	American Journal of Pathology	1983	129	3.39	Finland	Clinical
22	75	Symptomatic subependymoma: report of 21 cases with review of literature	Scheithauer BW	Journal of Neurosurgery	1978	141	3.28	USA	Clinical
91	76	Postoperative radiotherapy for intracranial ependymoma: analysis of prognostic factors and patterns of failure	Oya N, Hiraoka M	Journal of Neuro-Oncology	2002	62	3.26	Japan	Clinical
96	77	Astroblastoma: radiologic-pathologic correlation and distinction from ependymoma	Port JD, Pomper MG	American Journal of Neuroradiology	2002	59	3.11	USA	Clinical
85	78	Chromosome arm 6q loss is the most common recurrent autosomal alteration detected in primary pediatric ependymoma	Reardon DA, Look AT	Genes Chromosomes & Cancer	1999	65	2.95	USA	Basic science
97	79	Stereotactic radiosurgery for recurrent ependymoma	Stafford SL, Schomberg PJ	Cancer	2000	59	2.81	USA	Clinical
98	80	Pediatric low-grade and ependymal spinal cord tumors	Merchant TE, Kun LE	Pediatric Neurosurgery	2000	59	2.81	USA	Clinical
67	81	Intracranial ependymoma long-term outcome, patterns of failure	Kovalic JJ, Roth KA	Journal of Neuro-Oncology	1993	78	2.79	USA	Clinical
41	82	Subcutaneous sacrococcygeal myxopapillary ependymoma: a clinicopathologic study of 32 cases	Helwig EB, Stern JB	American Journal of Clinical Pathology	1984	102	2.76	USA	Clinical
93	83	Clinicopathologic study of 61 patients with ependymoma including mib-1 immunohistochemistry	Prayson RA	Annals of Diagnostic Pathology	1999	60	2.73	USA	Clinical
60	84	Ependymoma: internal correlations among pathological signs: the anaplastic variant	Schiffer D, Vigliani MC	Neurosurgery	1991	80	2.67	Italy	Clinical
62	85	The role of prophylactic spinal irradiation in localized intracranial ependymoma	Vanuytsel L, Brada M	International Journal of Radiation Oncology Biology Physics	1991	79	2.63	UK	Clinical
94	86	Survival following intensive chemotherapy with bone marrow reconstitution for children with recurrent intracranial ependymoma: a report of the children's cancer group	Mason WP, Finlay JL	Journal of Neuro-Oncology	1998	60	2.61	USA	Clinical
53	87	Ependymal and choroid-plexus tumors: cytokeratin and GFAP expression	Mannoji H, Becker LE	Cancer	1988	84	2.55	Canada	Clinical
68	88	Intracranial ependymoma and subependymoma: MR manifestations	Spoto GP, Solomon M	American Journal of Neuroradiology	1990	78	2.52	USA	Clinical
72	89	Intracranial ependymoma in children: analysis of prognostic factors	Chiu JK, Shallenberger R	Journal of Neuro-Oncology	1992	73	2.52	USA	Clinical
86	90	MR characteristics of histopathologic subtypes of spinal ependymoma	Kahan H, Bruce JH	American Journal of Neuroradiology	1996	63	2.52	USA	Clinical
99	91	Tanycytic ependymoma	Langford LA, Barre GM	Ultrastructural Pathology	1997	59	2.46	USA	Clinical
92	92	A high-dose busulfan-thiotepa combination followed by autologous bone marrow transplantation in childhood recurrent ependymoma: a phase-II study	Grill J, Hartmann O	Pediatric Neurosurgery	1996	61	2.44	France	Clinical
43	93	Extra-spinal ependymomas: report of 3 cases	Morantz RA, Masterson BJ	Journal of Neurosurgery	1979	99	2.36	USA	Clinical
20	94	A metastasizing ependymoma of the cauda equina	Weiss, L	Cancer	1955	148	2.24	USA	Clinical
48	95	Delayed distant metastasis from a subcutaneous sacrococcygeal ependymoma: case report, with tissue-culture, ultrastructural observations and review of literature	Wolff M, Duby MM	Cancer	1972	93	1.9	USA	Review
47	96	Secretory ependymoma of filum terminale	Miller CA Torack RA	Acta Neuropathologica	1970	95	1.86	USA	Clinical
51	97	Is subependymoma (subependymal glomerate astrocytoma) an astrocytoma or ependymoma: comparative ultrastructural and tissue-culture study	Fu YS, Young HF	Cancer	1974	86	1.83	USA	Clinical
87	98	Melanin as a component of cerebral gliomas: melanotic cerebral ependymoma	Mccloskey JJ, Blacker HM	Cancer	1976	63	1.4	USA	Clinical
61	99	Ependymoma of the brain: pathologic aspects	Svien HJ, Craig WM	Neurology	1953	80	1.18	USA	Review
71	100	A study of tumors arising from ependymal cells	Bailey P	Archives of Neurology And Psychiatry	1924	75	0.77	USA	Clinical

The most cited article overall was a basic science article entitled “Radial glia cells are candidate stem cells of ependymoma,” published in *Cancer Cell* in 2005 (Table 3) [51]. The second most cited article overall was a clinical article entitled “Intramedullary ependymoma of the spinal cord,” published in the *Journal of Neurosurgery* in 1990 (Table 4) [34]. Basic science and clinical articles comprised the majority of the top 50. The first review article ranked forty-second overall was titled “Pediatric ependymoma: biological perspectives” and was published in *Molecular Cancer Research* in 2009 (Table 1) [22].

**Table 3:** Most cited basic science articles on ependymoma

Basic science rank (TC)	Overall rank (TC)	Overall rank (CY)	Title	Authors (first/last)	Journal title	Publication year	Total citations	Average citations per year	Country
1	1	3	Radial glia cells are candidate stem cells of ependymoma	Taylor MD, Gilbertson RJ	Cancer Cell	2005	551	34.44	USA
2	3	1	Molecular classification of ependymal tumors across all CNS compartments, histopathological grades, and age groups	Pajtler KW, Pfister SM	Cancer Cell	2015	321	53.5	Germany
3	5	2	C11orf95-RELA fusions drive oncogenic Nf-kappa B signalling in ependymoma	Parker M, Gilbertson RJ	Nature	2014	272	38.86	USA
4	7	4	Delineation of two clinically and molecularly distinct subgroups of posterior fossa ependymoma	Witt H, Pfister SM	Cancer Cell	2011	244	24.4	Germany
5	9	7	Cross-species genomics matches driver mutations and cell compartments to model ependymoma	Johnson RA, Gilbertson RJ	Nature	2010	219	19.91	USA
6	10	25	Natural simian-virus-40 strains are present in human choroid-plexus and ependymoma tumors	Lednicky JA, Butel JS	Virology	1995	206	7.92	USA
7	14	14	Identification of tumor-specific molecular signatures in intracranial ependymoma and association with clinical characteristics	Modena P, Sozzi G	Journal of Clinical Oncology	2006	160	10.67	Italy
8	15	15	Identification of gains on 1q and epidermal growth factor receptor overexpression as independent prognostic markers in intracranial ependymoma	Mendrzyk F, Lichter P	Clinical Cancer Research	2006	156	10.4	Germany
9	16	29	Molecular genetic analysis of ependymal tumors: Nf2 mutations and chromosome 22q loss occur preferentially in intramedullary spinal ependymomas	Ebert C, Von Deimling A	American Journal of Pathology	1999	154	7	USA
10	18	34	Expression of vascular endothelial growth factor and its receptors in the anaplastic progression of astrocytoma, oligodendroglioma, and ependymoma	Chan AS, Chung LP	American Journal of Surgical Pathology	1998	151	6.57	Hong Kong
11	31	35	Erbb receptor signaling promotes ependymoma cell proliferation and represents a potential novel therapeutic target for this disease	Gilbertson RJ Ellison DW	Clinical Cancer Research	2002	123	6.47	USA
12	35	13	Molecular staging of intracranial ependymoma in children and adults	Korshunov A, Pfister SM	Journal of Clinical Oncology	2010	119	10.82	Germany
13	44	51	Chromosomal abnormalities subdivide ependymal tumors into clinically relevant groups	Hirose Y, Feuerstein BG	American Journal of Pathology	2001	98	4.9	USA
14	57	24	Identification of microRNAs as potential prognostic markers in ependymoma	Costa FF Soares MB	Plos One	2011	80	8	USA
15	63	20	A prognostic gene expression signature in infratentorial ependymoma	Wani K, Aldape K	Acta Neuropathologica	2012	78	8.67	USA
16	74	28	An integrated in vitro and in vivo high-throughput screen identifies treatment leads for ependymoma	Atkinson JM, Gilbertson RJ	Cancer Cell	2011	71	7.1	USA
17	78	49	Differential expression and prognostic significance of sox genes in pediatric medulloblastoma and ependymoma identified by microarray analysis	De Bont JM, Pieters R	Neuro-Oncology	2008	69	5.31	Netherlands
18	80	54	Ependymoma gene expression profiles associated with histological subtype, proliferation, and patient survival	Lukashova-Von Zangen I, Roggendorf W	Acta Neuropathologica	2007	66	4.71	Germany
19	85	78	Chromosome arm 6q loss is the most common recurrent autosomal alteration detected in primary pediatric ependymoma	Reardon DA, Look AT	Genes Chromosomes & Cancer	1999	65	2.95	USA

**Table 4:** Most cited clinical articles on ependymoma

Clinical rank (TC)	Overall rank (TC)	Overall rank (CY)	Title	Authors (first/last)	Journal title	Publication year	Total citations	Average citations per year	Country
1	2	8	Intramedullary ependymoma of the spinal cord	Mccormick PC, Stein BM	Journal of Neurosurgery	1990	453	14.61	USA
2	4	33	Ependymoma: follow-up-study of 101 cases	Mork SJ, Loken AC	Cancer	1977	290	6.59	Norway
3	6	6	Conformal radiotherapy after surgery for paediatric ependymoma: a prospective study	Merchant TE, Sanford RA	Lancet Oncology	2009	258	21.5	USA
4	8	38	Myxopapillary ependymoma: a clinicopathologic and immunocytochemical study of 77 cases	Sonneland PR, Onofrio BM	Cancer	1985	226	6.28	USA
5	11	12	Preliminary results from a phase II trial of conformal radiation therapy and evaluation of radiation-related CNS effects for pediatric patients with localized ependymoma	Merchant TE, Sanford RA	Journal of Clinical Oncology	2004	201	11.82	USA
6	12	40	The prognostic-significance of postoperative residual tumor in ependymoma	Healey EA, Tarbell NJ	Neurosurgery	1991	184	6.13	USA
7	13	21	Postoperative chemotherapy without irradiation for ependymoma in children under 5 years of age: a multicenter trial of the French society of pediatric oncology	Grill J, Kalifa C	Journal of Clinical Oncology	2001	173	8.65	France
8	17	44	Treatment of intracranial ependymomas of children: review of a 15-year experience	Rousseau P, Rey A	International Journal of Radiation Oncology Biology Physics	1994	154	5.7	France
9	19	63	Improved survival in cases of intracranial ependymoma after radiation-therapy: late report and recommendations	Salazar OM, Aygun C	Journal of Neurosurgery	1983	150	3.95	USA
10	20	94	A metastasizing ependymoma of the cauda equina	Weiss, L	Cancer	1955	148	2.24	USA
11	21	41	Analyses of prognostic factors in a retrospective review of 92 children with ependymoma: Italian Pediatric Neuro-oncology Group	Perilongo G, Madon E	Medical and Pediatric Oncology	1997	144	6	Italy
12	22	75	Symptomatic subependymoma: report of 21 cases with review of literature	Scheithauer BW	Journal of Neurosurgery	1978	141	3.28	USA
13	23	16	Primary postoperative chemotherapy without radiotherapy for intracranial ependymoma in children: the UKCCSG/SIOP prospective study	Grundy RG, Machin D	Lancet Oncology	2007	139	9.93	UK
14	24	74	Differential-diagnosis of chordoma, chondroid, and ependymal tumors as aided by anti-intermediate filament antibodies	Miettinen M, Virtanen I	American Journal of Pathology	1983	129	3.39	Finland
15	25	57	Intracranial ependymoma: long-term results of a policy of surgery and radiotherapy	Vanuytsel LJ, Brada M	International Journal of Radiation Oncology Biology Physics	1992	128	4.41	UK
16	26	32	Spinal cord ependymoma: radical surgical resection and outcome	Hanbali F, Gokaslan ZL	Neurosurgery	2002	127	6.68	USA
17	27	17	Proton radiotherapy for childhood ependymoma: initial clinical outcomes and dose comparisons	Macdonald SM, Yock T	International Journal of Radiation Oncology Biology Physics	2008	126	9.69	USA
18	28	47	Ependymoma: results, prognostic factors and treatment recommendations	Mclaughlin MP, Million RR	International Journal of Radiation Oncology Biology Physics	1998	126	5.48	USA
19	29	69	Postoperative radiotherapy of intracranial ependymoma in pediatric and adult patients	Shaw EG, Earle JD	International Journal of Radiation Oncology Biology Physics	1987	126	3.71	USA
20	30	11	Histopathological grading of pediatric ependymoma: reproducibility and clinical relevance in European trial cohorts	Ellison DW, Grundy RG	Journal of Negative Results in Biomedicine	2011	123	12.3	USA
21	32	26	Monomorphous angiocentric glioma: a distinctive epileptogenic neoplasm with features of infiltrating astrocytoma and ependymoma	Wang M, Burger PC	Journal of Neuropathology and Experimental Neurology	2005	121	7.56	USA
22	33	46	A multi-institutional retrospective study of intracranial ependymoma in children: identification of risk factors	Horn B, Russo C	Journal of Pediatric Hematology Oncology	1999	121	5.5	USA
23	34	61	Identification of a germ-line mutation in the p53 gene in a patient with an intracranial ependymoma	Metzger AK, Cogen PH	Proceedings of The National Academy of Sciences of the United States of America	1991	120	4	USA
24	36	45	Combined postoperative irradiation and chemotherapy for anaplastic ependymomas in childhood: results of the German prospective trials hit 88/89 and hit 91	Timmermann B Bamberg M	International Journal of Radiation Oncology Biology Physics	2000	118	5.62	Germany
25	37	65	Histologic prognostic factors in ependymoma	Schiffer D, Tribolo A	Childs Nervous System	1991	116	3.87	Italy
26	38	19	Incidence patterns for ependymoma: a surveillance, epidemiology, and end results study clinical article	Mcguire CS, Fisher PG	Journal of Neurosurgery	2009	112	9.33	USA
27	39	59	Adjuvant chemotherapy of childhood posterior fossa ependymoma: cranio-spinal irradiation with or without adjuvant CCNU, vincristine, and prednisone: a children’s cancer group study	Evans AE, Finlay JL	Medical and Pediatric Oncology	1996	106	4.24	USA
28	40	73	Postoperative radiotherapy in the management of spinal-cord ependymoma	Whitaker SJ, Brada M	Journal of Neurosurgery	1991	105	3.5	UK
29	41	82	Subcutaneous sacrococcygeal myxopapillary ependymoma: a clinicopathologic study of 32 cases	Helwig EB, Stern JB	American Journal of Clinical Pathology	1984	102	2.76	USA
30	43	93	Extra-spinal ependymomas: report of 3 cases	Morantz RA, Masterson BJ	Journal of Neurosurgery	1979	99	2.36	USA
31	45	60	Adjuvant chemotherapy for the treatment of intracranial ependymoma of childhood	Needle MN, Phillips PC	Cancer	1997	97	4.04	USA
32	46	27	A retrospective study of surgery and reirradiation for recurrent ependymoma	Merchant TE, Sanford RA	International Journal of Radiation Oncology Biology Physics	2008	96	7.38	USA
33	47	96	Secretory ependymoma of filum terminale	Miller CA Torack RA	Acta Neuropathologica	1970	95	1.86	USA
34	51	97	Is subependymoma (subependymal glomerate astrocytoma) an astrocytoma or ependymoma: comparative ultrastructural and tissue-culture study	Fu YS, Young HF	Cancer	1974	86	1.83	USA
35	52	71	Anaplastic ependymoma: treatment of pediatric patients with or without craniospinal radiation therapy	Merchant TE, Leibel SA	Journal of Neurosurgery	1997	85	3.54	USA
36	53	87	Ependymal and choroid-plexus tumors: cytokeratin and GFAP expression	Mannoji H, Becker LE	Cancer	1988	84	2.55	Canada
37	54	37	Predicting change in academic abilities after conformal radiation therapy for localized ependymoma	Conklin HM, Merchant TE	Journal of Clinical Oncology	2008	83	6.38	USA
38	55	31	Both location and age predict survival in ependymoma: a seer study	Mcguire CS, Fisher PG	Pediatric Blood & Cancer	2009	81	6.75	USA
39	56	72	Treatment of intracranial ependymoma by surgery alone	Hukin J, Allen J	Pediatric Neurosurgery	1998	81	3.52	USA
40	59	48	Spinal myxopapillary ependymoma outcomes in patients treated with surgery and radiotherapy at MD Anderson Cancer Center	Akyurek S, Woo SY	Journal of Neuro-Oncology	2006	80	5.33	USA
41	60	84	Ependymoma: internal correlations among pathological signs: the anaplastic variant	Schiffer D, Vigliani MC	Neurosurgery	1991	80	2.67	Italy
42	62	85	The role of prophylactic spinal irradiation in localized intracranial ependymoma	Vanuytsel L, Brada M	International Journal of Radiation Oncology Biology Physics	1991	79	2.63	UK
43	64	42	Multifactorial analysis of predictors of outcome in pediatric intracranial ependymoma	Ridley L, Grundy RG	Neuro-Oncology	2008	78	6	UK
44	65	52	Radiation dosimetry predicts iq after conformal radiation therapy in pediatric patients with localized ependymoma	Merchant TE, Mulhern RK	International Journal of Radiation Oncology Biology Physics	2005	78	4.88	USA
45	66	53	Ependymoma: new therapeutic approaches including radiation and chemotherapy	Merchant TE, Fouladi M	Journal of Neuro-Oncology	2005	78	4.88	USA
46	67	81	Intracranial ependymoma long-term outcome, patterns of failure	Kovalic JJ, Roth KA	Journal of Neuro-Oncology	1993	78	2.79	USA
47	68	88	Intracranial ependymoma and subependymoma: MR manifestations	Spoto GP, Solomon M	American Journal of Neuroradiology	1990	78	2.52	USA
48	69	18	Proton radiotherapy for pediatric central nervous system ependymoma: clinical outcomes for 70 patients	Macdonald SM, Yock TI	Neuro-Oncology	2013	76	9.5	USA
49	70	50	Human telomere reverse transcriptase expression predicts progression and survival in pediatric intracranial ependymoma	Tabori U, Hawkins C	Journal of Clinical Oncology	2006	76	5.07	Canada
50	71	100	A study of tumors arising from ependymal cells	Bailey P	Archives of Neurology And Psychiatry	1924	75	0.77	USA
51	72	89	Intracranial ependymoma in children: analysis of prognostic factors	Chiu JK, Shallenberger R	Journal of Neuro-Oncology	1992	73	2.52	USA
52	73	66	Ependymoma in childhood: prognostic factors, extent of surgery, and adjuvant therapy	van Veelen-Vincent, ML, Renier D	Journal of Neurosurgery	2002	72	3.79	Netherlands
53	75	67	Influence of tumor grade on time to progression after irradiation for localized ependymoma in children	Merchant TE, Kun LE	International Journal of Radiation Oncology Biology Physics	2002	71	3.74	USA
54	76	68	Preliminary results from a phase II trial of conformal radiation therapy for pediatric patients with localized low-grade astrocytoma and ependymoma	Merchant TE, Kun LE	International Journal of Radiation Oncology Biology Physics	2002	71	3.74	USA
55	77	58	The high incidence of tumor dissemination in myxopapillary ependymoma in pediatric patients: report of five cases and review of the literature	Fassett DR, Kestle JRW	Journal of Neurosurgery	2005	70	4.38	USA
56	79	62	A multicenter study of the prognosis and treatment of adult brain ependymal tumors	Reni M, Villa E	Cancer	2004	68	4	Italy
57	81	64	Ki-67 immunolabeling index is an accurate predictor of outcome in patients with intracranial ependymoma	Wolfsberger S, Hainfellner J	American Journal of Surgical Pathology	2004	66	3.88	Austria
58	82	9	Clinical evidence of variable proton biological effectiveness in pediatric patients treated for ependymoma	Peeler CR, Grosshans DR	Radiotherapy and Oncology	2016	65	13	USA
59	83	10	Therapeutic impact of cytoreductive surgery and irradiation of posterior fossa ependymoma in the molecular era: a retrospective multicohort analysis	Ramaswamy V, Taylor MD	Journal of Clinical Oncology	2016	65	13	Canada
60	84	43	Primary postoperative chemotherapy without radiotherapy for treatment of brain tumours other than ependymoma in children under 3 years: results of the first UKCCSG/SIOP CNS 9204 trial	Grundy RG, Machin D	European Journal of Cancer	2010	65	5.91	UK
61	86	90	MR characteristics of histopathologic subtypes of spinal ependymoma	Kahan H, Bruce JH	American Journal of Neuroradiology	1996	63	2.52	USA
62	87	98	Melanin as a component of cerebral gliomas: melanotic cerebral ependymoma	Mccloskey JJ, Blacker HM	Cancer	1976	63	1.4	USA
63	88	30	Survival benefit for pediatric patients with recurrent ependymoma treated with reirradiation	Bouffet E, Tabori U	International Journal of Radiation Oncology Biology Physics	2012	62	6.89	Canada
64	89	56	Outcome for young children newly diagnosed with ependymoma, treated with intensive induction chemotherapy followed by myeloablative chemotherapy and autologous stem cell rescue	Zacharoulis S, Finlay J	Pediatric Blood & Cancer	2007	62	4.43	USA
65	90	70	Hyperfractionated radiotherapy and chemotherapy for childhood ependymoma: final results of the first prospective aieop (Associazione Italiana di Ematologia-Oncologia Pediatrica) study	Massimino M, Madon E	International Journal of Radiation Oncology Biology Physics	2004	62	3.65	Italy
66	91	76	Postoperative radiotherapy for intracranial ependymoma: analysis of prognostic factors and patterns of failure	Oya N, Hiraoka M	Journal of Neuro-Oncology	2002	62	3.26	Japan
67	92	92	A high-dose busulfan-thiotepa combination followed by autologous bone marrow transplantation in childhood recurrent ependymoma: a phase-II study	Grill J, Hartmann O	Pediatric Neurosurgery	1996	61	2.44	France
68	93	83	Clinicopathologic study of 61 patients with ependymoma including mib-1 immunohistochemistry	Prayson RA	Annals of Diagnostic Pathology	1999	60	2.73	USA
69	94	86	Survival following intensive chemotherapy with bone marrow reconstitution for children with recurrent intracranial ependymoma: a report of the children's cancer group	Mason WP, Finlay JL	Journal of Neuro-Oncology	1998	60	2.61	USA
70	96	77	Astroblastoma: radiologic-pathologic correlation and distinction from ependymoma	Port JD, Pomper MG	American Journal of Neuroradiology	2002	59	3.11	USA
71	97	79	Stereotactic radiosurgery for recurrent ependymoma	Stafford SL, Schomberg PJ	Cancer	2000	59	2.81	USA
72	98	80	Pediatric low-grade and ependymal spinal cord tumors	Merchant TE, Kun LE	Pediatric Neurosurgery	2000	59	2.81	USA
73	99	91	Tanycytic ependymoma	Langford LA, Barre GM	Ultrastructural Pathology	1997	59	2.46	USA
74	100	23	Clinical, radiological, histological and molecular characteristics of paediatric epithelioid glioblastoma	Broniscer A, Ellison DW	Neuropathology and Applied Neurobiology	2014	58	8.29	USA

**Fig. 1:**
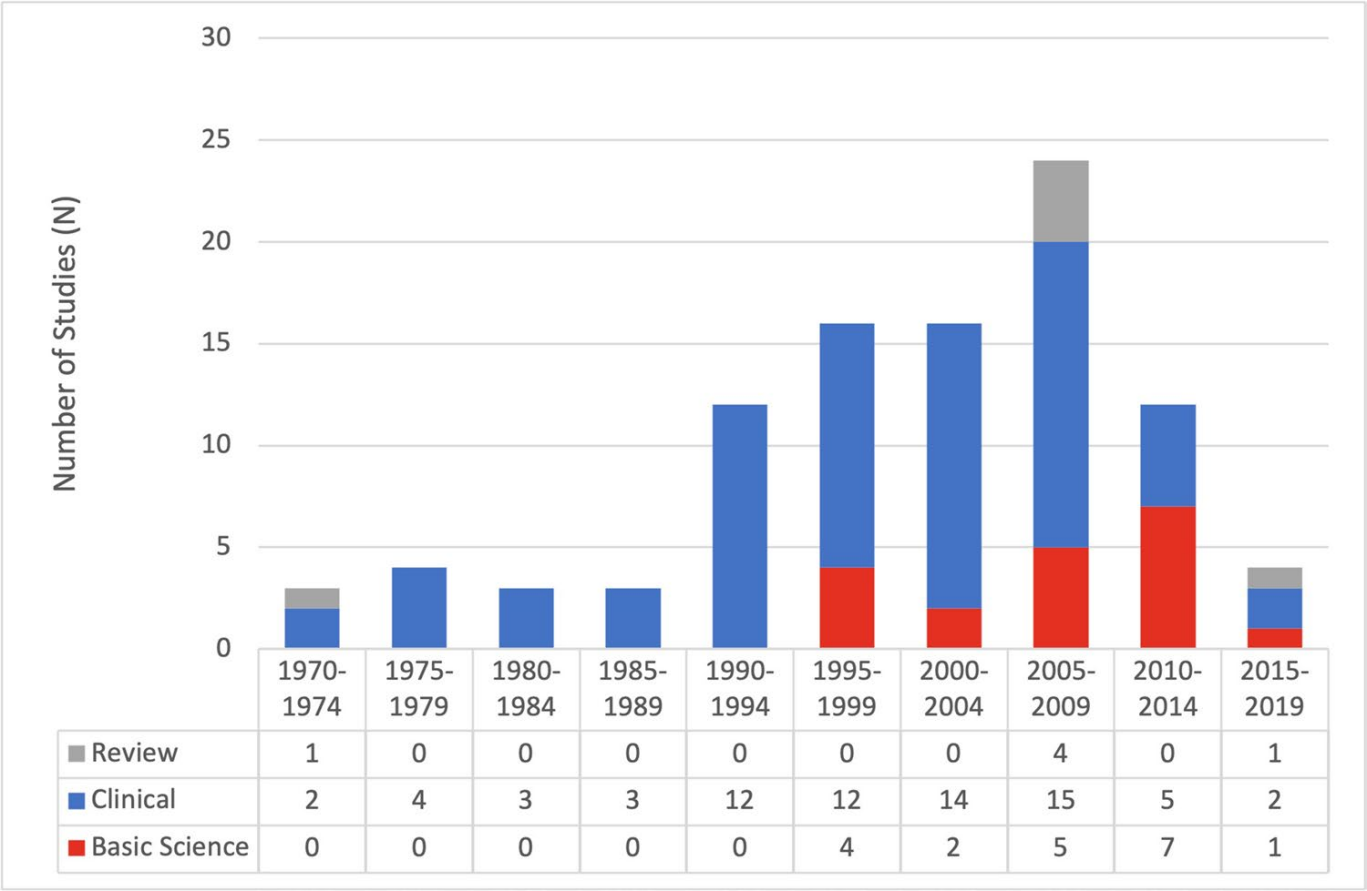
Proportion of clinical, basic science, and review articles that were published over each 5-year period, starting in 1970

**Fig. 2:**
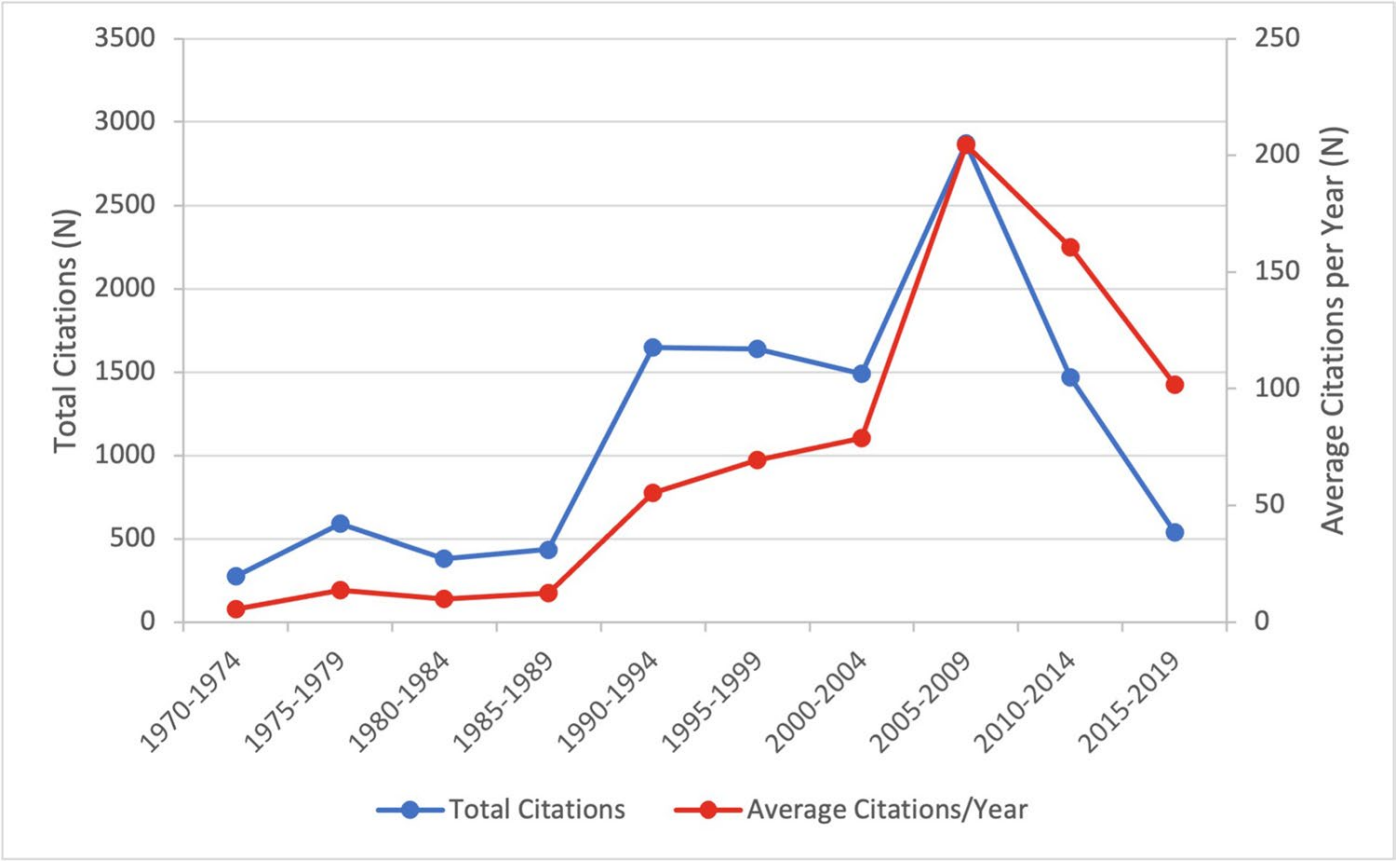
Total citations and average citations per year for articles that were published over each 5-year period

As shown in Fig. 1, the time period from 2005 to 2009 oversaw the publication of the greatest number of articles on the list (24 papers). This was followed by 2000–2004 and 1995–1999 (16 papers each) (Fig. 1). Total citations (2870) and average citations per year (204) were also highest for papers published in 2005–2009 (Fig. 2).

### Journal of publication

The top 100 cited articles on ependymoma were published in 35 unique journals. The most frequent journals featuring the top cited articles included *International Journal of Radiation Oncology Biology Physics* (13%), *Cancer* (10%), and *Journal of Neurosurgery* (9%) (Table 5). Of the top 10 most cited, 3 articles were published in *Cancer Cell*, followed by 2 articles each in *Nature* and *Cancer*.

**Table 5:** Number of articles per journal

Journals of publication	Number of articles (n = 100)
International Journal of Radiation Oncology Biology Physics	13
Cancer	10
Journal of Neurosurgery	9
Journal of Clinical Oncology	7
Journal Of Neuro-Oncology	6
Acta Neuropathologica	4
Cancer cell	4
Neuro-Oncology	4
American Journal of Neuroradiology	3
American Journal of Pathology	3
Neurosurgery	3
Pediatric Neurosurgery	3
American Journal of Surgical Pathology	2
Clinical Cancer Research	2
Journal of Neuropathology and Experimental Neurology	2
Lancet Oncology	2
Medical and Pediatric Oncology	2
Nature	2
Pediatric Blood & Cancer	2
Other *	17

^*^Journals with one article on the top 100 list

### Countries and institutions

**Fig. 3:**
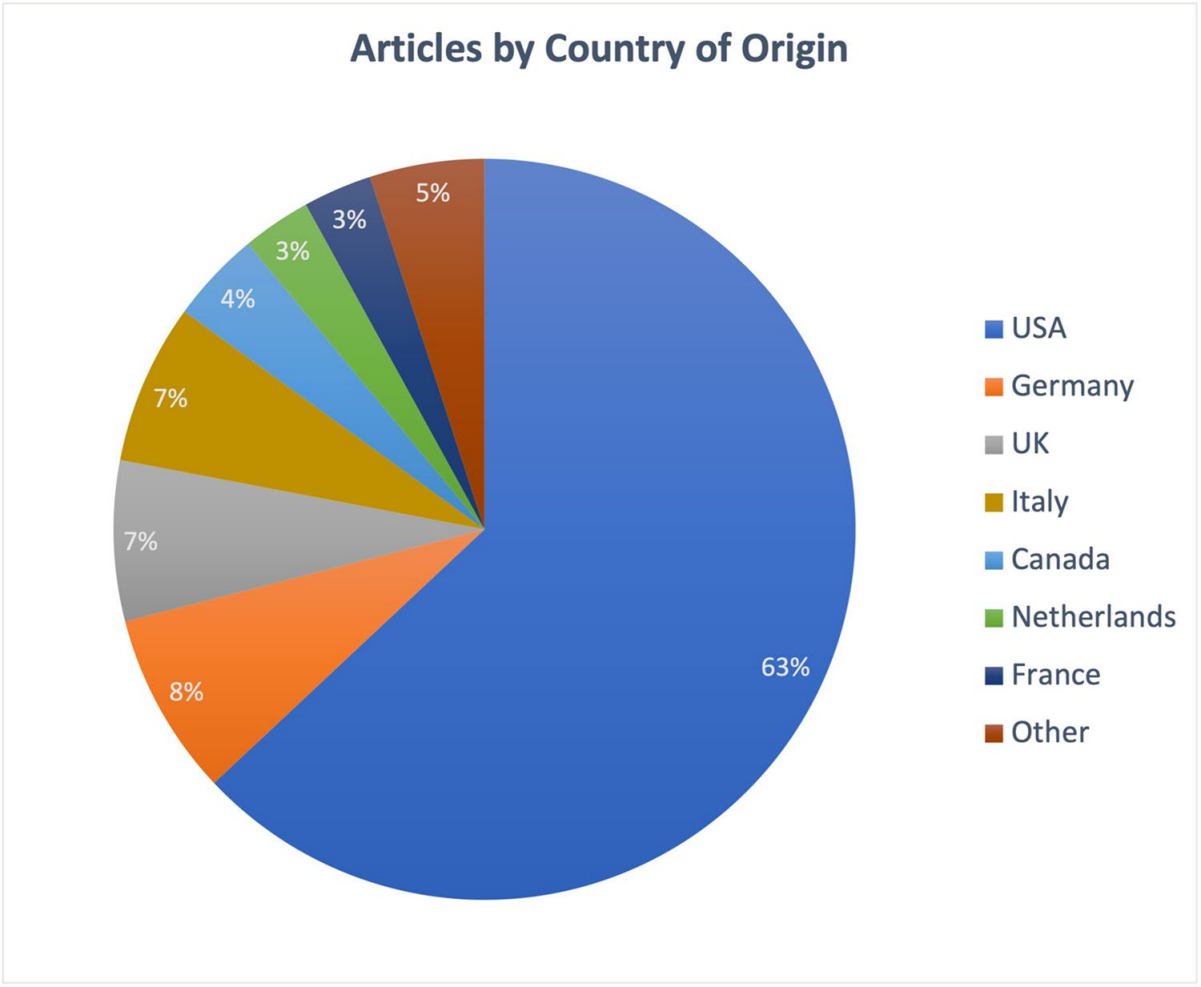
Proportion of articles coming from each country of origin. The category “other” includes Japan, Austria, Hong Kong, Finland, and Norway, each of which had 1 article

A total of 13 countries represented the top 100 articles published (Fig. 3). The USA (n = 63), Germany (n = 8), and the UK (n = 7) were the highest contributors of the top 100 articles. The top institutions contributing the greatest number of articles among the top 100 most cited articles were St. Jude Children’s Research Hospital (n = 16), the University of Texas MD Anderson Cancer Center (n = 6), and the German Cancer Research Center (n = 5) (Table 6). The USA contributed 5 of the top 10 most cited articles.

**Table 6:** Top institutions (based on first author)

Institution	Country	Number of articles
St Jude Children’s Research Hospital	USA	16
The University of Texas MD Anderson Cancer Center	USA	6
German Cancer Research Center	Germany	5
Hospital for Sick Children	Canada	4
Mayo Clinic	USA	4
Stanford University	USA	4
Royal Marsden Hospital	UK	3
University of Nottingham	UK	3
Children’s Hospital of Philadelphia	USA	2
Institut Gustave Roussy	France	2
Istituto Nazionale Tumori	Italy	2
Memorial Sloan-Kettering Cancer Center	USA	2
San Raffaele Scientific Institute	Italy	2
Sophia Children’s Hospital	Netherlands	2
University of California, San Francisco	USA	2
Washington University School of Medicine	USA	2
University of Turin	Italy	2

### Article category

Each article was categorized as either basic science (19%), clinical (74%), or literature review (7%) (Table 1; Fig. 1). Studies are separated into basic science and clinical studies and ranked by times cited in Tables 3 and 4, respectively. Of the top 10 articles, 6 were basic science articles and 4 were clinical articles. Of the top 20, 10 were basic science articles, and 10 were clinical articles.

### Citations per year

Since articles published more remotely are advantaged in terms of collecting citations over time, we examined the citation frequency per year. Using this metric, the article with the greatest number of citations per year (53.5) was a basic science article entitled “Molecular classification of ependymal tumors across all CNS compartments, histopathological grades, and age groups,” published in *Cancer Cell* in 2015 (Table 2) [42]. Comparatively, the clinical article with the most citations per year — “Conformal radiotherapy after surgery for pediatric ependymoma: a prospective study,” published in *Lancet Oncology* in 2009 — averaged far fewer (21.5) (Table 2) [35].

### Authors

**Fig. 4:**
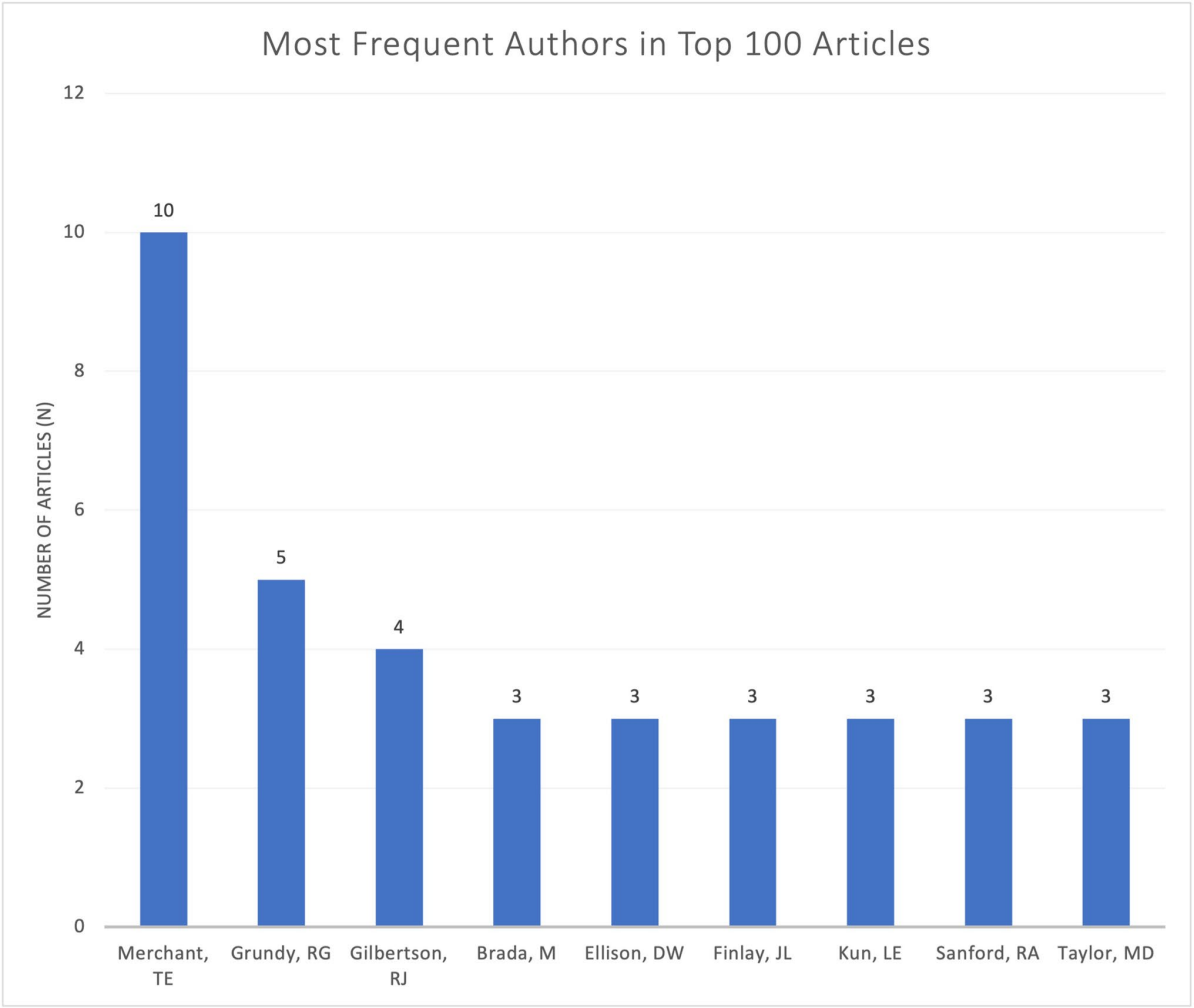
Number of articles published by the most common authors based on presence as either first or last author in the top 100 most cited articles

The first and senior authors of each paper in the top 100 list were analyzed (Table 1). Thomas E. Merchant from St Jude Children’s Research Hospital authored the greatest number of articles (10), followed by Richard G. Grundy (5) from Children’s Brain Tumour Research Centre, University of Nottingham, and Richard J. Gilbertson (4) from St Jude Children’s Research Hospital (Fig. 4).

## Discussion

This study identifies the most widely cited articles related to the understanding of ependymoma. Our bibliometric analysis revealed 100 articles published across 35 distinct journals, which highlighted a broad international interest in ependymoma research. While a large majority of the top 100 cited articles were clinical (74%), basic science research (19%) comprised half of the top 20 most cited articles. This is likely the result of a recent focus on novel molecular classifications for the disease, as well as an effort to better understand the biochemical underpinnings of its development to guide therapeutic strategies. The large volume of literature focused on ependymoma research can pose a challenge for anyone searching for significant, impactful studies in the field [17]. Our hope is that this bibliometric analysis informs researchers in their efforts to understand the most relevant and significant literature relating to ependymoma.

It is important to note that while overall citation number is an important indicator of an article’s impact and importance, it can be misleading in older articles that have more time to be cited with each passing year. To account for this, our analysis included another important metric: citations per year (Table 2). As an example, the article ranked sixth overall on our list — “Conformal radiotherapy after surgery for pediatric ependymoma: a prospective study” published in *Lancet Oncology* in 2009 — also ranked sixth in citations per year [35]. This article reported a high rate of local tumor control and event-free survival following aggressive surgical intervention and adjuvant high-dose conformal radiotherapy in pediatric patients, including those younger than 3 years of age [35]. Its presence within the top 10 in both overall and average yearly citations indicates its continued relevance in our understanding of ependymoma, particularly for pediatric patients, despite having been published over ten years ago.

In bibliometric analyses, it is not uncommon to find several articles with drastically different positions on these two lists. Such articles tend to be highly impactful articles published very recently. Two such articles on our list worth examining in closer detail are studies by Peeler et al. and Ramaswamy et al. in 2016. Ranked 81st and 82nd overall and 9th and 10th in citations per year, respectively, these two studies provided novel insights into two well-established treatment modalities. Specifically, Peeler et al. discovered that proton therapy-induced damage to normal tissue dependent on the physical radiation dose and track-averaged linear energy transfer, one of the main determinants of proton therapy’s biological effectiveness [45]. Ramaswamy et al. reported that incomplete resection of molecular variant EPN_PFA (posterior fossa ependymoma A) ependymomas was associated with poor prognosis and that adjuvant radiation is preferred for patients with complete resections, while delayed external-bean radiation is preferred for relapsing cases of EPN_PFB (posterior fossa ependymoma B) tumors [46]. Both articles highlight critical discoveries in our understanding of current therapeutics for ependymoma, so it is unsurprising that they have each been given considerable attention since publication. Their place on the overall citation list is likely just a consequence of having less time to gather citations.

A closer examination of the top 20 articles in particular revealed a trend with respect to article type and publication year. Clinical articles within the top 20 tended to be published earlier (i.e., 1955 to 2009), while basic science articles tended to be published later (i.e., 1995 to 2015). Logically, the basic science articles in the top 20 had higher average citations per year (21.4) than clinical articles (8.7). These clinical articles tended to focus on the initial clinical presentations and pathophysiologic prognosticators of the disease, much of which is considered common knowledge today. One such article, entitled “Ependymoma: follow-up study of 101 cases,” published in *Cancer* in 1977 (fourth most cited overall), managed to follow a cohort of patients who underwent ependymoma treatment over a considerable period of time (22 years) [37]. The authors reported favorable clinical outcomes in cases of spinal ependymoma, which more commonly affects adults (10-year survival of 72%), compared to intracranial ependymoma, which more commonly affects children (10-year survival of 13%) [37]. They also reported a survival benefit with postoperative radiation therapy but failed to find much prognostic value in tissue histopathology, an issue still under debate in current literature [27, 52, 57, 59]. Given the extensive follow-up reported by the authors as well as the relatively novel findings with respect to clinical course of ependymoma at the time of publication (1977), it is not surprising that this clinical article has maintained citation prevalence to date. The article entitled “Myxopapillary ependymoma: a clinicopathologic and immunocytochemical study of 77 cases,” published in *Cancer* in 1985 and eighth overall on our list, is another example of a clinical article that has maintained relevance despite its remote publication date [50]. This study focused on gross tumor characteristics as prognosticators for postoperative course, reporting that certain physical findings, such as the presence of a tumor capsule, were more indicative of prognosis than histological features [50]. Since the publication of these and other similar clinical articles, advances in biomolecular research have improved our understanding of the molecular underpinnings of ependymomas. Such advances have likely contributed to the recent shift in focus from ependymoma’s clinical characteristics and prognostic factors to biomolecular properties of the disease. Continued scientific interest in ependymoma molecular biology, technological advancements, and new innovations may eventually give rise to novel treatments, such as small molecule and personalized precision medicine therapies [39, 44, 54].

The top cited clinical studies on ependymoma are most often case series describing key clinical features, diagnostic modalities, different treatment regimens, and outcomes. One common theme among studies is that GTR is the single factor most consistently associated with improved survival and reduced recurrence compared to subtotal resection (STR) [15, 18, 35, 36, 47, 50]. There were no prospective randomized controlled trials in the top 100 most cited articles. The most cited clinical article (ranked second overall) — titled “Intramedullary ependymoma of the spinal cord,” was published in 1990 and described a retrospective series of 23 patients who underwent surgical resection of this entity [34]. All tumors were histologically benign, gross total resection was achieved in all cases, and no recurrences were reported. Other series reported outcomes in various treatment strategies combining surgical resection, radiation therapy, and chemotherapy. For instance, the 19th most cited study (by Salazar et al. published in the *Journal of Neurosurgery* in 1983) was one of the first studies to establish efficacy of adjunctive radiotherapy in ependymoma treatment [48]. The authors reported a 10-year overall survival of 69% in a series of patients with intracranial ependymoma treated with resection and whole-brain radiation therapy [48]. The progression from whole-brain radiation to localized radiation was demonstrated in a more recent prospective trial by Merchant et al. in 2009. These authors published a large series of 153 pediatric patients who underwent surgery and conformal radiation therapy (CRT) and reported 85% overall survival in patients who received CRT without delay [35]. In addition to their excellent outcomes, this study irradiated pediatric patients younger than 3 years old, which has been historically avoided due to concerns for delayed radiation neurotoxicity [16]. The role of adjunctive chemotherapy in ependymoma treatment was the topic of two prospective trials that were 13th [15] and 23rd [16] most cited studies overall. The 13th most cited study involved treatment of 73 children with primarily high-grade ependymoma with surgery and chemotherapy, without radiation [15]. The authors reported a low 4-year progression-free survival rate at 22% and overall survival rate of 59% [15]. The 23rd most cited study treated 89 children aged 3 years or younger with surgical resection and chemotherapy. Similarly, disease progression occurred in 62.5% of patients with non-metastatic disease, and overall survival at 5 years was 63.4% [16]. Notably, the authors did report that higher doses of chemotherapy were associated with improved 5-year overall survival compared to low doses (76% vs. 52%) [16].

Several clinical studies were lower in overall citations but higher in citations per year, suggesting that they are impactful articles published more recently. For instance, the study entitled —“Histopathological grading of pediatric ependymoma: reproducibility and clinical relevance in European trial cohorts,” published in 2011 was 30th in overall citations but 11th in citations per year [11]. This study developed a novel method for ependymoma grading that demonstrated higher concordance among pathologists than the traditional WHO grading method. However, the study found little correlation between ependymoma grade and clinical outcomes, calling into question the clinical utility of histological grading of ependymoma [11]. Two studies ranked 9th [45] and 17th [26] in average citations per year, utilized proton beam radiation for adjunctive ependymoma treatment. Peeler et al. created linear regression models correlating proton beam radiation dose and linear energy transfer with post-treatment changes on imaging. This demonstrated objective clinical changes caused by proton beam radiation, although did not report patient outcomes such as overall or progression-free survival [45]. On the other hand, MacDonald et al. reported excellent 2-year overall survival (89%) and progression-free survival (80%) in 17 pediatric patients treated with proton therapy after surgical resection. These studies together may represent a promising new adjunct to GTR in the treatment of ependymoma. Finally, as previously mentioned, one study ranked 83rd overall and 10th by citations per year addressed the effect of distinct molecular profiles of posterior fossa ependymoma on outcomes after surgery and radiation [46]. The authors report EPN_PFA was a highly significant predictor of poor progression-free survival (hazard ratio [HR], 2.14; 95% confidence interval [CI], 1.31 to 3.49, *P* = 0.002) and overall survival (HR, 4.30; 95% CI, 1.88 to 9.87; *P* < 0.001). Conversely, EPN_PFB was associated with excellent 10-year overall survival of 96.1% after GTR [46]. These findings in this recent article with a high citations per year count highlight the new appreciation of ependymoma molecular subtyping in treatment prognosis.

The WHO grading criteria for ependymoma based on tumor histopathology (most recently updated in 2016) have been shown to have poor predictive value for overall survival for the disease [27]. Given the limited clinical utility of these criteria, recent research has focused on understanding the molecular biology of ependymoma to improve on our current prognostic capabilities [27]. Six of the 10 most cited articles were basic science studies aimed at addressing the issue of ependymoma subtyping. The top article overall, entitled “Radial glia cells are candidate stem cells of ependymoma,” published in *Cancer Cell* in 2005, found that supratentorial, infratentorial, and spinal cord ependymomas are derived from radial glial cells [51]. From this, the authors suggested that histologically similar ependymomas from different regions of the central nervous system represent molecularly distinct diseases and that ependymomas have gene expression profiles that resemble regionally specific radial glial cells. More recently, the article entitled “C11orf95-RELA fusions drive oncogenic NF-kappa B signalling in ependymoma” published in *Nature* in 2015 elaborated upon the genetic underpinnings of a well-known oncogenic pathway (NF-κB; nuclear factor kappa-light-chain-enhancer of activated B cells), which was found to exist in two-thirds of supratentorial ependymomas [43]. Subsequently, a RELA fusion-positive (grade II or III) ependymoma subtype was included in the 2016 WHO Classification of Tumors of the CNS [12].

Another 2015 study focusing on ependymal classification — “Molecular classification of ependymal tumors across all CNS compartments, histopathological grades, and age groups” published in *Cancer Cell* — ranked third overall and first in citations per year [42]. This study used DNA methylation profiling to identify nine distinct molecular subgroups of ependymoma and subcategorized each according to its location within the CNS (supratentorial, posterior fossa, and spine) [42]. The novel predictive system developed by this study outperformed previously published histopathological classifications in predicting overall and progression-free survival. The DNA-methylation-specific categorization was not included in the 2016 WHO Classification of Tumors of the CNS likely because DNA methylation profiling is only available in restricted institutions [23] and is therefore not amenable to widespread implementation [41]. Collectively, the recent momentum favoring biomolecular research in ependymoma has led to a more robust classification system for the disease, which will allow for improved prognostication and narrowed molecular targeting for therapeutic development. Such advancements are imperative given the high (40%) prevalence of incurable tumors, poor postoperative prognosis, and chemotherapy-resistant properties of ependymomas [19, 43, 51]. Continued research will reveal the impact of these basic science investigations on the therapeutic and diagnostic landscape of these tumors.

### Limitations

This study has several limitations. First, as previously discussed, our list of the top 100 most cited papers was generated based on the total number of citations, which is subject to bias towards papers published earlier [3, 10]. Conversely, more recently published articles are often shown more frequently in research databases, which may also contribute to bias. To address this issue, we included data on the total number of citations (Table 1) and average citations per year in our analysis (Table 2), in order to provide a comprehensive view of ependymoma research. This analysis also demonstrated that basic science articles have enjoyed more citations on average in the last 10 years than clinical articles. Taken in combination with overall citation data, these findings suggest that the current direction of ependymoma research will focus more heavily on research examining the biomolecular characteristics of ependymoma. Second, while WoS is the most commonly used and validated resource for bibliometric analyses, it is not comprehensive of all medical literature and does not include citations from textbooks or non-English journal articles [10, 17]. Our WoS search was also title-specific, which may have led to the unintentional exclusion of relevant papers in the top 100 list since abstracts and full-text articles were not included in the search. Third, bibliometric analyses carry the inherent limitation that the citation frequency does not always correlate with impact. For instance, a basic science article published in 2016 on childhood posterior fossa ependymomas published in *Science Translational Medicine* determined that reduced H3K27me3 and DNA hypomethylation were associated with poor clinical outcomes [5]. However, this impactful study did not make the top 100 list. Fourth, our list is subject to inaccuracy due to the phenomenon of “obliteration by incorporation,” whereby highly important articles can become less frequently cited over time as their ideas or findings become so widely accepted as to be considered common knowledge (and thus cited anonymously) [33]. As such, citation numbers may not always accurately reflect the influence or impact of studies, a limitation that is not completely addressed despite our using previously validated bibliometric analysis methodologies for CNS tumors [3, 17, 25]. Fifth, we categorized studies as basic science and clinical based on the focus of each article as previously performed [25]. However, this dichotomization did not account for studies that may be further subclassified as translational in nature. Despite these limitations, this article seeks to present publishing trends within the ependymoma literature and provides a categorized reference of articles and synthesis that will be helpful for future clinical trainees and scientists in the neuro-oncological and neurosurgical fields.

## Conclusion

This study used a validated bibliometric analysis to identify the top 100 most cited articles on ependymoma. Careful examination of the list, in conjunction with another important metric — average number of citations per year — helps paint a picture of the history and behavior of ependymoma research over the last 50 years, as its focus migrated from clinical correlates and histopathologic prognosticators to genetic and molecular underpinnings of the disease. That we observe a high proportion of recently published basic science articles in the top 20 papers of our list points to a tendency to improve upon what were once widely accepted histopathological grading criteria. Ependymomas are chemotherapy-resistant, and a large proportion of tumors are incurable even with surgery and radiotherapy. Our results suggest that the field of ependymoma research is moving towards a more robust basic biological understanding and molecular classification system to guide clinical decision-making and future research endeavors into potential therapeutic options.

## Data Availability

All data and materials support published claims and comply with field standards.
